# Supplementary material for: Imaging and mapping the impact of clouds on skyglow with all-sky photometry
Source: Sci Rep. 2017 Jul 27;7:6741. doi: 10.1038/s41598-017-06998-z (PMC5532222; doi:10.1038/s41598-017-06998-z)
Supplement: Supplementary file 1 — Supplementary Information [file 41598_2017_6998_MOESM1_ESM.pdf]

Supplemental Information

Imaging and mapping the impact of clouds on skyglow with all-sky photometry

Andreas Jechow[1,2], Zoltán Kolláth[3], Salvador J. Ribas[4,5], Henk Spoelstra[6], Franz Hölker[1], Christopher C. M. Kyba[2,1]

[1] *Ecohydrology, Leibniz Institute of Freshwater Ecology and Inland Fisheries, Müggelseedamm 310, 12587 Berlin*
[2] *Remote Sensing, Helmholtz Center Potsdam, German Center for Geosciences GFZ, Telegraphenberg, Potsdam*
[3] *Eötvös Loránd University, Savaria Department of Physics, Károlyi Gáspár tér 4, 9700 Szombathely, Hungary*
[4] *Parc Astronmic Montsec, Comarcal de la Noguera, Pg. Angel Guimer 28-30, 25600 Balaguer, Lleida, Spain*
[5] *Institut de Cincies del Cosmos (ICCUB), Universitat de Barcelona, C.Mart i Franqus 1, 08028 Barcelona, Spain and*
[6] *LightPollutionMonitoring.Net, Urb. Veïnat Verneda 101 (Bustia 49), 17244 Cassà de la Selva, Girona, Spain*

The supplement contains the full data set of all-sky luminance maps obtained at the 8 locations along the transect on the two nights. The figures S1 and S2 show the clear night sky data on the left column and the overcast night sky data on the right column. Each row represents a location at different distances form the town of Balaguer, Spain.

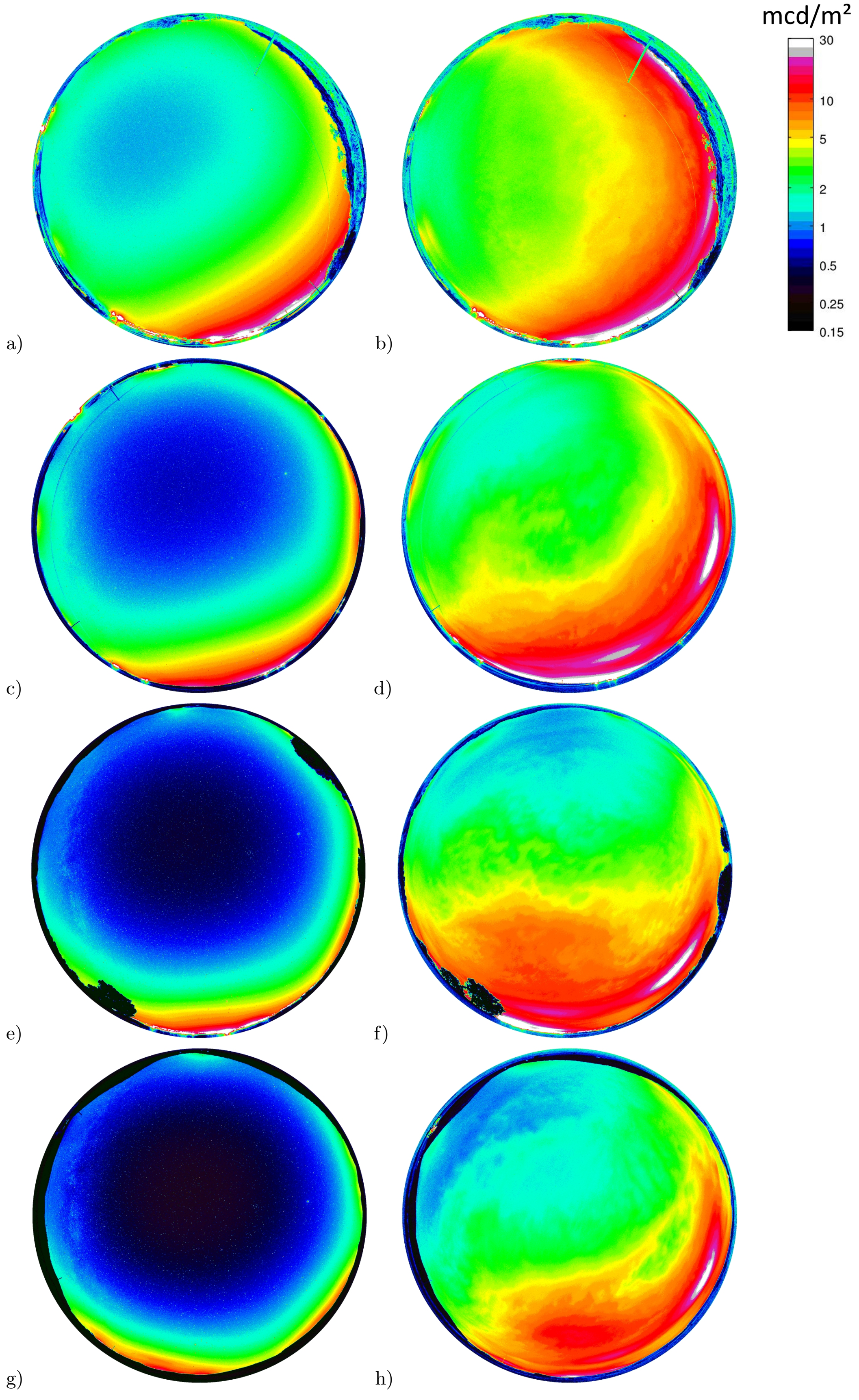

FIG. S1: All-sky luminance maps along the transect for clear (a, c, e, g) and partly cloudy night (b, d, f, h). The upper row (a, b) was acquired at 1.1 km distance, (c, d) at 2.2 km distance, (e, f) at 4.8 km distance and (g, h) 8.4 km distance to the city center of Balaguer, Spain.

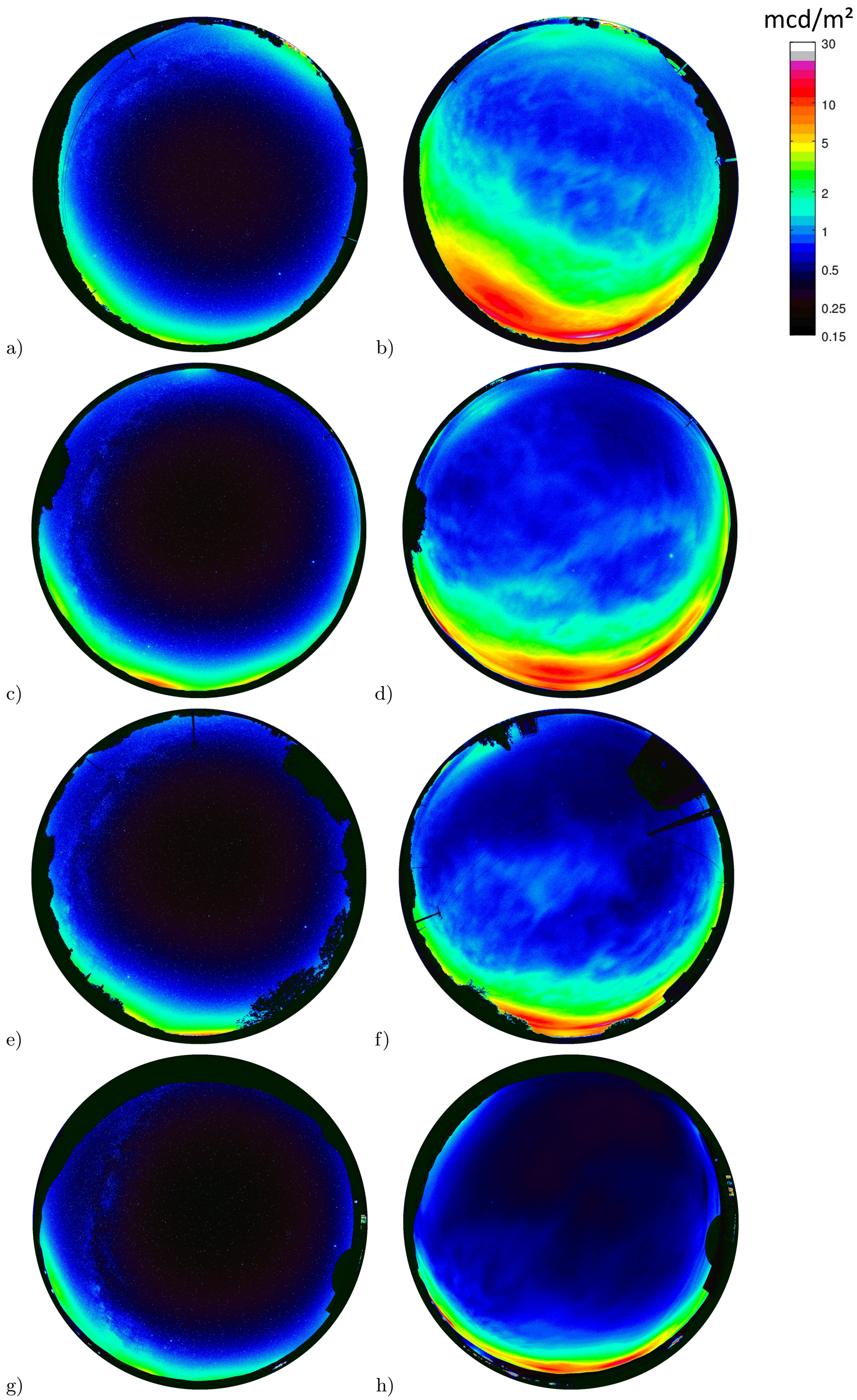

FIG. S2: All-sky luminance maps along the transect for clear (a, c, e, g) and partly cloudy night (b, d, f, h). The upper row (a, b) was acquired at 13.5 km distance, (c, d) at 17.7 km distance, (e, f) at 22.2 km distance and (g, h) at PAM at 27.2 km distance to the city center of Balaguer, Spain.
